# *In silico* evaluation, characterization, and *in vitro* anticancer activity of curcumin–nimbin loaded nanoformulation in HCT-116 cell lines

**DOI:** 10.5114/bta.2024.145256

**Published:** 2024-12-19

**Authors:** Arumugam Madeswaran, Selvam Tamilazhagan, Sellappan Mohan

**Affiliations:** Department of Pharmacology, Karpagam College of Pharmacy, affiliated to the Tamil Nadu Dr. M.G.R. Medical University, Coimbatore, Tamil Nadu, India

**Keywords:** nanosuspension, inhibition constant, MTT assay, binding energy

## Abstract

Colorectal cancer is one of the most prevalent malignancies worldwide and a leading cause of mortality. Chemotherapy medications are often limited in use due to issues like drug resistance, P-glycoprotein efflux, and relapse of chemotherapy. In this study, we formulated a nanosuspension with curcumin and nimbin to address these limitations and assessed its anticancer potential using *in silico* molecular docking and *in vitro* MTT assay. Methods. *In silico* docking and ADMET analyses targeted proteins implicated in colorectal cancer, with doxorubicin as the standard. The docking studies were conducted using AutoDock 4.2, while *in vitro* anticancer activity was assessed through the MTT assay in HCT 116 cell lines. Results. *In silico* docking of curcumin and nimbin showed significant interactions with target proteins compared to the standard. ADMET analysis indicated favorable Caco-2 permeability and intestinal absorption of the selected phytoconstituents. The MTT assay demonstrated concentration-dependent cell viability inhibition in HCT 116 cell lines treated with the nanosuspension, with an IC_50_ value of 30%. Conclusion. The curcumin–nimbin loaded nanosuspension demonstrated promising anticancer activity against HCT 116 cell lines in both *in silico* and *in vitro* studies. Further studies are required to evaluate the anticancer effect of curcumin–nimbin loaded nanosupension through clinical and preclinical studies for the progress of potential formulation in the treatment of colorectal cancer.

## Introduction

Most cancers are due to infection in which many of the body compartments are elevated wildly and spread to diverse portions of the body. Cancer might be a bland period for an expansive gathering of ailments that can disturb any part of the body. Additional phrases developed are hazardous tumors and neoplasms (Dekker et al., 2019).

Colorectal cancer (CRC) is a common, lethal, and preventable disorder. CRC is the second main motive of cancer in both sexes, in the range of 10–11% of cancer deaths. The clinical direction of colon cancer is generally silent and first of all without signs until the disorder has reached an advanced level. A good way to find cancerous or precancerous lesions at an early, curable degree is a complete video-colonoscopy is usually recommended at 5–12 months intervals, for any individual 30 years and up (Guren, 2019).

For patients with family records of colon cancer or colon polyps, an every-year screening is counseled. Also, most people do now not follow those recommendations, putting themselves prone to dying of colon cancer or dwelling with bothersome colostomy for the rest of their lives. One of the fundamental purposes of the colonoscopy is the screening for polyps. Polyps are small or medium-sized tumors that develop without signs and symptoms some of them can also evolve into cancer (Schreuders et al., 2015).

Turmeric is a spice that has acquired a whole lot of interest from each of the clinical/scientific worlds in addition to from the culinary international. Curcumin, turmeric’s active ingredient, has been known to have medical properties for thousands of years, but just now researchers have been able to pinpoint the exact mode of action and recognize their bioactive principles (Rabeneck et al., 2020). A triterpenoid called nimbin was discovered from the neem. Numerous biological effects of neem oil are thought to be attributed to nimbin, which is also thought to have anti-inflammatory, antipyretic, fungicidal, antihistamine, and antiseptic properties. A chemical from the Neem tree known as nimbin may be found in several Asian countries, including China, Thailand, and India. The chemical family of limonoids and triterpenoids includes nimbin (Priyadarsini, 2014).

Doxorubicin is an antineoplastic inside the anthracycline magnificence. Most of those compounds had been remoted from herbal resources and antibiotics. The anthracyclines are most of the maximum vital antitumor tablets to be had. Doxorubicin is broadly used for the remedy of numerous strong tumors while idarubicin and daunorubicin are used solely for the management of leukemia. Doxorubicin may additionally prevent the action of polymerase, have an effect on gene expression regulation, and convey loose impairment in the free radical to DNA (Susan and Douglas, 2017).

*In silico* drug design refers to rational drug discovery and design utilizing computer techniques. Drug likeness quantifies the likelihood that a chemical will develop into an oral medication in terms of bioavailability. Due to the early evaluation of ADMET qualities during medication development, a considerable decrease in the compounds that were unsuccessful in the clinical trials due to subpar pharmacokinetic properties has occurred (Jose et al., 2012). Due to its many uses in the drug development process, molecular docking – protein – ligand interaction is the subject of most research. Typically, the ligand is a tiny chemical that interacts with the binding sites of the target protein (Kubinyi, 1999).

Nanosuspensions are colloidal diffusions of medication elements that are submicron in size and surfactants are used for stabilization. Nanosuspensions include drugs that are poorly soluble in both lipid and water platforms and are suspended in dispersion without the need for a matrix material. These suspensions can be utilized to improve the solubility of medications. The rate of flooding of the active component rises and the maximum plasma level is achieved faster as a result of prolonged solubility. This method is helpful for compounds that provide a significant challenge for formulators due to their low solubility, horrible permeability, or both. The particle size reduction helps to manage poorly soluble capsules intravenously without causing any capillary obstruction (Kapetanovic, 2008).

The KRAS proto-oncogene in HCT116 cells has an alteration at codon 13, creating suitable transfection targets for experiments involving gene therapy. The cells exhibit an epithelial appearance, are capable of metastasizing in xenograft models, and persist in the G1 phase after being introduced with viral vectors expressing the p53 gene. Five-Fu/P85 copolymer micelles were shown to limit the growth of HCT116 cell lines. Furthermore, it was shown that stress in the endoplasmic reticulum produced by the removal of MARCH 2 prevented the growth of HCT116 cells (Lakshmi and Ashwini, 2010). Hence the objective of the study is to evaluate the curcumin and nimbin nanosuspension for its anticancer property using *in silico* and *in vitro* MTT assay.

## Materials and methods

Software used. PubChem for procuring of ligands, RCSB database for purification of proteins, pkCSM for evaluation of drug-likeness properties and Pharmacokinetic (ADMET) prediction, SPDB viewer for preparation of target proteins, BIOVIA Discovery studio for optimization of ligands and clustering, PyRx for *in silico* docking process and PyMOL for constructing the protein ligand complex.

Chemicals used. Curcumin, nimbin, doxorubicin, beta-cyclodextrin, sodium lauryl sulfate, methanol, non-absorbent cotton, rotary evaporator, pH meter, mechanical stirrer, ultra-sonication, UV-visible spectrophotometer, malvern zeta seizer, fourier transform infrared (FT-IR) spectroscopy.

### Evaluation of drug-likeness properties

Lipinski’s rule of 5 is dependent on five straight-forward physiochemical restriction arrays: the molecular weight which should not be more than 500 Daltons, the lipophilicity should be less than five, less than five hydrogen bond donors and less than ten hydrogen bond acceptors, and the topological polar surface area, which should be less than 140, respectively (Xia, 2017; Lipinski et al., 1997).

### Prediction of ADMET properties of ligands

ADMET analyses of selected compounds were predicted using pkCSM software. This online platform predicts the pharmacokinetic parameters of the unknown ligands. The selected compounds were delivered to the platform and the results were tabulated and analyzed (Madeswaran et al., 2017).

### Docking interaction analysis

The target molecule K-RAS (Kirsten rat sarcoma viral oncogene homolog) [1FKY] was refined by removing the water molecules and unwanted ligand residues and adding the hydrogen molecules in the Spdb viewer. The refined target molecule was saved as a.pdb file which was further used for docking studies. The selected ligand molecules such as curcumin and nimbin were prepared using the Biovia Discovery Studio viewer. The energy minimization of the ligand was carried out using PyRx software. The grid box was fixed in the geometric center of the target molecule. The docking was carried out using PyRx software. The binding orientation and the binding parameters of the selected ligand molecules were evaluated against the target molecule (Mun et al., 2022).

### Methods for extraction and formulation

#### Plant material

The seeds of *Azadirachta indica* were procured from the campus of Karpagam College of Pharmacy, Coimbatore. The plant was authorized by the Botanical Survey of India, Tamil Nadu Agriculture University Campus, Coimbatore, Tamil Nadu. The authentication number for *Azadirachta indica* is BSI/SRC/5/23/2022/Tech/649. Curcumin (5 g) was purchased from Sigma Aldrich, Bangalore. The separated neem seed kernels were crushed and powdered and used for the present study.

### Extraction and isolation of nimbin

300 g of seed kernels were obtained from ripe neem seeds were extracted after they were crushed. Then it was pulverized and defatted in hexane (500 ml, three times) for 12 h. Then, using an overhead mechanical stirrer, 1.5 l of methanol was extracted from the defatted neem kernels for 12 h. To get around 70 g of crude extract, the extracted solvent was sieved and intense under decreased pressure. The EtOAc layer was detached and intense after partitioning with ethyl acetate (100 × 3 ml) and water to produce 30 g of final crude extract. This was once again executed using a hexane and ethyl acetate solvent combination in silica gel column chromatography (100–120 mesh). 600 mg of nimbin (N1) and the required nimbin (Hexanes: EtOAc) eluted at around 70 : 30 ratio (Asokkumar et al., 2012).

### Polymeric-based curcumin–nimbin loaded nanosuspension

25 mg of curcumin and 25 mg of nimbin were thoroughly dissolved in 20 ml of methanol, which was immediately added to 50 ml of distilled water containing diverse concentrations of Sodium lauryl sulfate (SLS) and Beta-cyclodextrin (bCD). This process took place under the influence of sonication (40 kHz; Lark, India). However, to eliminate any remaining methanol from the Nano suspension, sonication was continued for an additional 60 min. Centrifugation was used to separate the SLS/bCD mixed curcumin–nimbin loaded nanoparticles (Remi, India) at 19 000 rpm for approximately 45 min at −20°C . They were cleaned and re-suspended in distilled water (Megalai and Syed, 2016).

### Characterization of curcumin–nimbin loaded nanosuspension

#### UV-Visible spectral analysis

1 ml of the mixture was exposed periodically and their spectrums were measured with a UV-visible spectrophotometer in the range of 200–800 nm (Moorthi et al., 2013).

#### Particle size analysis

Size and size distribution of the prepared polymericbased curcumin–nimbin loaded nanosuspension were obtained by a Zetasizer (Nano ZS; Malvern Instruments Ltd, UK) using the dynamic light scattering (DLS) method (Moorthi et al., 2013). In each measurement, 1.0 ml of the sample was distributed in a square polystyrene cuvette (DTS0012; Malvern, UK). Also, the zeta potential of the surface of the curcumin-nimbin-loaded nanosuspension was determined with the same instrument by folded capillary cells (DTS1070; Malvern, UK). Zete-potential and size were evaluated as the average of three separate capacities (Siavashy et al. 2021).

### Fourier transforms infrared spectroscopic (FTIR) analysis

For FTIR analysis, a pellet was created by combining 2 mg of the powdered nanoparticle sample with 20 mg of KBr. The sample holder was filled with the pellet, and the FTIR spectrum of polymeric-based curcumin–nimbin loaded nanosuspension was recorded in the range between 4000–500 cm^-1^ (Megalai and Syed, 2016).

### In vitro cytotoxicity assay

#### Cell culture

Epithelial human HCT cell lines were acquired from NCCS, Pune and cultured in Dulbecco’s Modified Eagle Medium (DMEM) augmented with 10% Fetal Bovine Serum (FBS), 1% sodium pyruvate 1% L-glutamine, and 1% sodium bicarbonate while being kept at 37°C and 80% humidity (Mohamed et al., 2022).

### MTT assay 3-(4,5-dimethylthiazol-2-yl)-2,5-diphenyltetrazolium bromide

Using HCT cells, a 3-(4,5-dimethylthiazol-2-yl)-2,5-diphenyltetrazolium bromide (MTT) assay was executed to evaluate the prepared nanoformulation *in vitro* cytotoxicity. Briefly, trypsinization was performed to produce the cultivated HCT cells, which were collected in a 15 ml tube. Following that, the cells were spread onto 96-well plates in DMEM media for 24–48 h at 37°C. The wells were cleaned with sterile PBS before being exposed to different sample attentions in a serum-free DMEM medium. The cells were cultured for 24 h at 37°C in a 5% CO_2_ incubator, with each sample being replicated three times. After the incubation, MTT (100 l of 0.5 mg/ml) was poured into each well and the cells were then let to continue incubating for an additional 2–4 h until purple precipitates were visible below a reversed microscope. Finally, 100 l of the medium containing MTT was enunciated from the wells and splashed with PBS. Additionally, DMSO (100 l) was added, and the plate was disturbed for 5 min to soften the formazan crystals. A microplate reader was used to quantify the absorbance for each well at 570 nm and compute the percentage of cell viability (Vinod et al., 2020).

### Statistical analysis

GraphPad was used for the statistical analysis of the data, which consisted of a one-way analysis of variance followed by Dunnett’s test. Results were expressed as mean ± standard deviation (SD). A *P*-value < 0.05 was considered statistically significant.

## Results

### Evaluation of drug-likeness

In the evaluation of drug likeness curcumin showed one violation and nimbin resulted in two violations whereas the standard drug exhibited four violations. Curcumin and nimbin showed better results than the standard drug. Hence, the above-selected compounds obeyed the Lipinski rule of five.

### ADMET prediction of ligands

The results of the ADMET predictions exhibited the good permeability and low toxic profile of curcumin and nimbin when compared to the standard doxorubicin. The selected compounds exhibited better options for the management of anticancer drugs.

### Docking interaction analysis

**Fig. 1:**
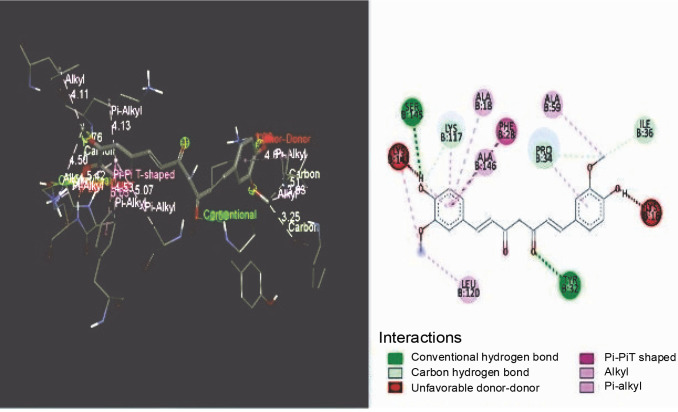
Binding orientations of curcumin against KRAS

The constituents such as curcumin, nimbin, and doxorubicin were docked with the selective target such as K-RAS. The protein conveyed signals to the cell’s nucleus from sources outside of the cell. These signals provided the cell commands for the progression and proliferation of the specialized roles. The BIOVIA Discovery studio visualizer tool was used to display the binding orientations. As illustrated in Figure 1, the curcumin was joined to proteins using two standard hydrogen bonds, such as SER B: 145, TYR B: 32, and three carbon-hydrogen bonds, such as LYS B: 117, PRO B: 34, and ILE B: 36.

**Fig. 2:**
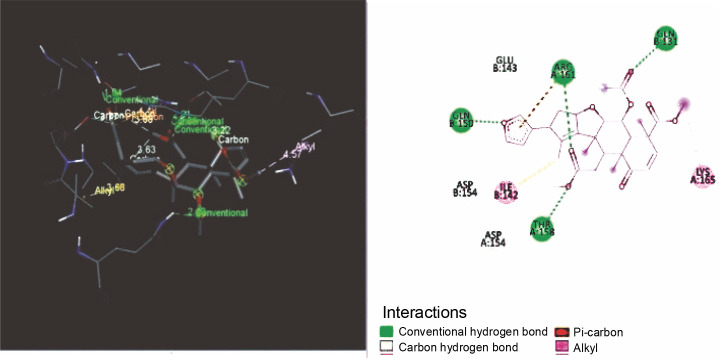
Binding orientations of nimbin against KRAS

The nimbin was bound to proteins with four conventional hydrogen bonds such as ARG A: 161, GLN B: 131, THR A: 158, and GLN B: 150, and three carbon–hydrogen bonds GLU B: 143, ASP A: 154, ASP B: 154 shown in Figure 2.

**Fig. 3:**
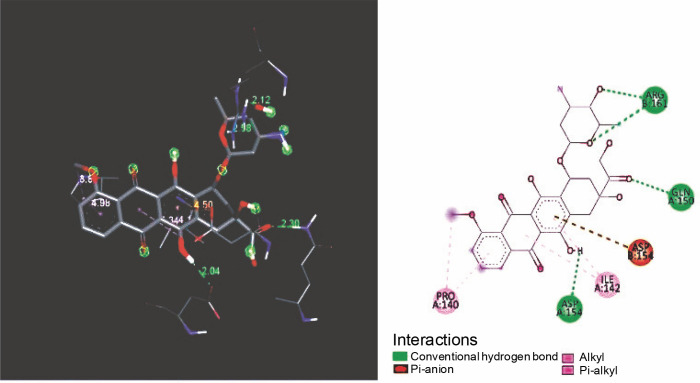
Binding orientations of doxorubicin against KRAS

The standard doxorubicin was bonded to proteins with three conventional hydrogen bonds GLU B: 143, ASP A: 154, ASP B: 154, and two alkyl groups ILE B: 142, LYS A: 165 were highlighted in Figure 3.

From the docking studies, it was perceived that the curcumin and nimbin exhibited noteworthy interaction with the KRAS target protein molecule when compared to that of the standard doxorubicin. The compounds curcumin and nimbin resulted in docking score values of −8.20 kcal/mol and −7.60 kcal/mol respectively. The standard compound doxorubicin produced a docking score of −10.30 kcal/mol against KRAS.

Early drug discovery predictions of pharmacokinetic features were crucial in directing hit-to-lead and leadoptimization efforts (Manikandan et al., 2012). The Lipinski rule of five ligands predicts a molecule as a medication for use topically or orally. Turmeric (*Curcuma longa*) root contains a phytopolyphenol molecule called curcumin, which is naturally occurring and has a variety of biological benefits. This study aimed to assess the purported antiproliferative, wound healing, anti-invasive, and antimigratory properties of curcumin on HCT-116 and colorectal cancer cell lines (Mar et al., 2018).

In the present study, bioactive compounds such as curcumin and nimbin were used as potential ligand molecules and the *in silico* docking studies were performed after initial screening. The *in silico* molecular docking studies revealed that the selected compounds possess anticancer activity.

### UV-Visible Spectral Analysis

For preliminary validation of the synthesis of polymeric-based, curcumin-nimbin loaded nanoparticles in nanosuspension with their surface plasmon resonance was characterized by UV-visible spectroscopy. A time-dependent UV-visible analysis was performed to evaluate the reaction’s duration. The absorption peak was shown to have grown from 270 to 430 nm.

**Fig. 4:**
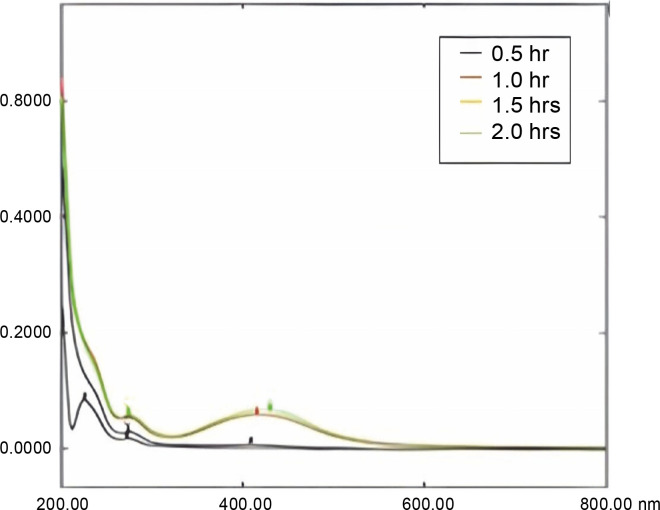
Time-dependent UV-visible absorbance spectra by UV for polymeric-based CUR–NIM loaded nanosuspension

Surface Plasmon Resonance (SPR) wavelength dramatically elevated when the reaction time was increased. The process of the development of surface plasmon peaks was observed at 425 nm at room temperature. It represented the confirmation of the synthesis of curcumin-loaded nanoparticles on a polymeric basis. The peaks were observed as narrow with a timeline, resulting in monodispersed particles (Fig. 4).

This finding is dependable with the absorption peak of curcumin-loaded nanoparticles exhibiting a prominent absorption peak at 425 nm. This comparison in absorption limits designates that curcumin was captured in the prepared nanoformulation (Wang et al., 2018). It recommends that the chemical composition of curcumin endured intact, with no substantial structural changes.

### FTIR analysis

**Fig. 5:**
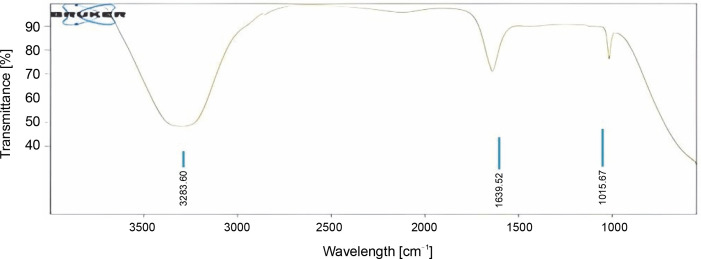
FTIR polymeric-based CUR–NIM loaded nanosuspension

The results revealed information about the surface chemistry of curcumin and nimbin. The obtained peaks indicated the presence of functional groups such as C=O, C–O, C–H & O–H groups. The surface chemistry of the functional groups involved in capping, reducing, and stabilizing the polymeric-based CUR–NIM-loaded nanosuspension (Fig. 5).

The peaks of the pure sample of curcumin was observed at 3506.46, 1626.57, 1601.18, 1505.79, 1427.38, 1315.42, 1273.17, 1231.99, 1203.98, 1181.75, 1151.40, 1114.14, 1025.30, 977.65, 961.68, 885.37, 855.77, 807.70, and 714.16 cm^-1^ and nimbin peaks were observed at 3327.00, 2939.72, 2832.31, 1603.97, 1399.09, and 1020.28 cm^-1^. The polymeric-based curcumin–nimbin loaded nanosuspension peaks were observed at 3283.60, 1639.52, and 1015.67 cm^-1^.

These findings are reliable with previous studies by Rompicharla et al. (2017) and Hettiarachchi et al. (2021) which also described the presence of these functional groups in curcumin. The presence of these representative functional groups in the prepared formulation endorses that there were no significant structural alterations in the curcumin compound. Therefore, the compound can be measured in nanoformulation for *in vitro* experiments and preserves its biological characteristics.

After loading of curcumin and nimbin with suitable polymers its absorption bands exhibited the common functional groups such as C=O, C–H, and C–O stretching vibrations of IR spectrum observed at 3283.60, 1639.52, and 1015.67 cm^-1^. The IR peaks directed that prepared nanoformulation was stable. Hence, it could be identified that the presence of carbonyl compounds, aliphatic hydrocarbons, and ketones in the prepared nanoformulation.

### Physiochemical characterization

**Fig. 6:**
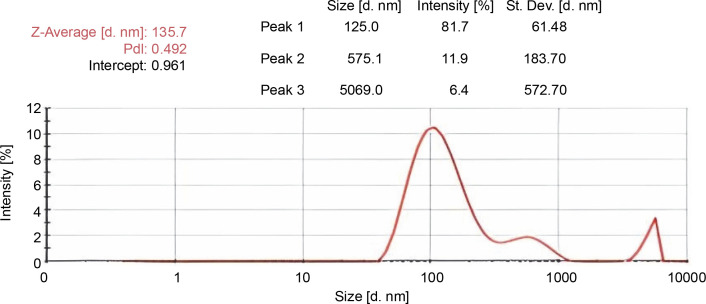
Particle size CUR–NIM loaded nanoparticles

The particle size analysis was done by dynamic light scattering which exposed the size distribution of synthesized polymeric-based CUR–NIM loaded nanoparticles. The average particle size was found to be 135.7 diameters value in nanometer (d. nm) and the polydispersity index (PDI) value was identified as 0.492 (Fig. 6).

**Fig. 7:**
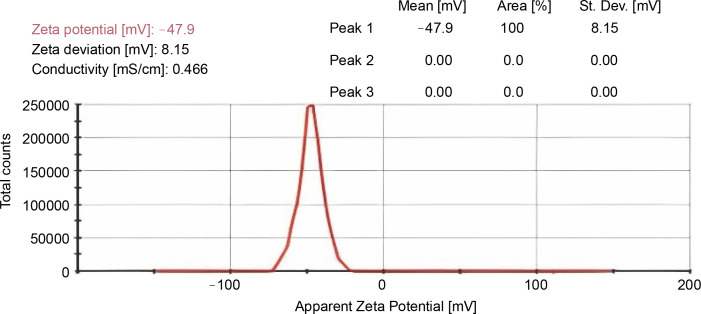
Zeta potential of CUR–NIM loaded nanoparticles

Zeta potential (ZP) is a physical characteristic that is revealed by any particle in suspension, material surface, or macromolecule. It can be used to augment the prepared formulations. In this study, the ZP value of the prepared nanoformulation was found to be −47.9 mV and the zeta deviation was identified as 8.17 mV (Fig. 7).

Literature has emphasized that the size of nanoparticles disturbs membrane permeability and tissue distribution of the synthesized drug molecule (Islam et al., 2017). Due to their increase in drug loading capacity, bulky surface area, controlled drug release, and reduction in particle size, nanoformulations have confirmed a significant impression on the management and prognosis of numerous diseases for *in vivo* studies (Choi et al., 2007). Therefore, the results of the present study suggest that the smaller particle size, significant PDI value, and ZP of the prepared curcumin nimbin nanoformulation make it appropriate for further studies.

### In-vitro MTT assay

**Fig. 8:**
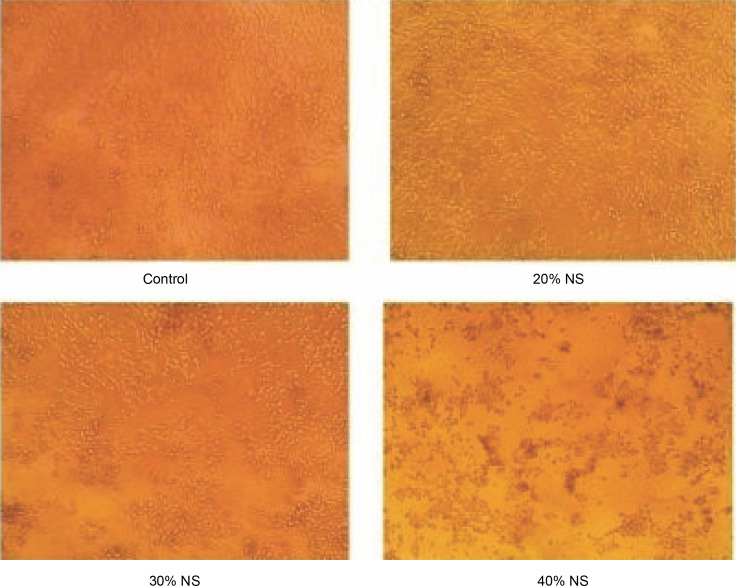
Pictorial representation of Control vs HCT 116 cell lines treated with curcumin–nimbin loaded nanosuspension

**Fig. 9:**
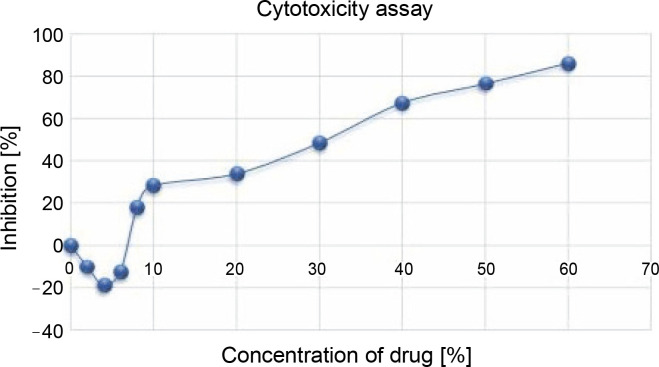
HCT 116 cell lines treated with curcumin–nimbin loaded nanosuspension

The anticancer activity of the prepared Nano formulation was evaluated using the MTT assay using HCT 116 cell lines and the results, as shown in Figure 8, demonstrated that the curcumin nimbin nanoformulation significantly inhibited the growth of HCT-116 cells compared to the control (untreated). The percentage of inhibition of curcumin nimbin nanoformulation showed a concentration-dependent manner. The IC_50_ of the given nanosuspension was found to be 30% (Fig. 9).

**Fig. 10:**
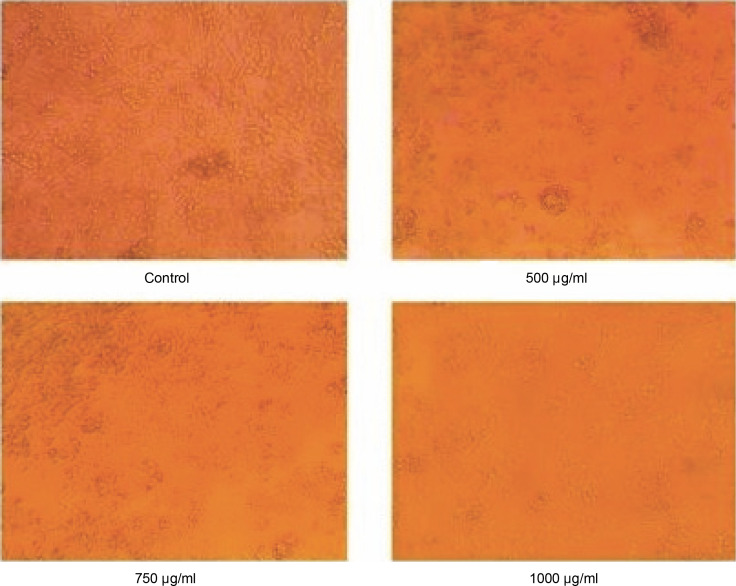
Pictorial representation of Control vs HCT 116 cell lines treated with standard doxorubicin

**Fig. 11:**
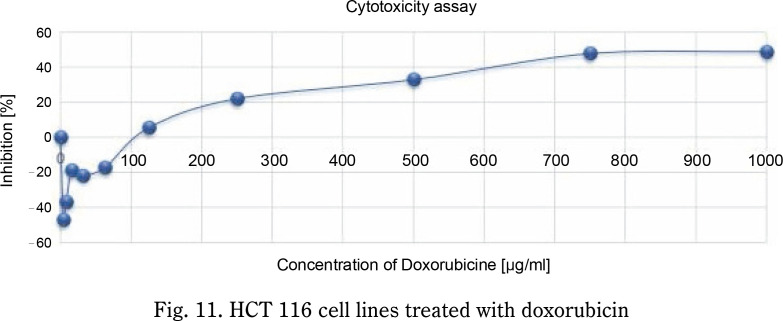
HCT 116 cell lines treated with doxorubicin

The anticancer activity of the standard doxorubicin was shown in Figure 10, the standard significantly inhibited the growth of HCT-116 cells compared to the untreated control. The percentage of inhibition of the standard drug resulted in a concentration-dependent manner. The IC_50_ of the given standard drug was found to be 750 μg/ml (Fig. 11).

Mukherjee and Vishwanatha prepared the nanospheres of curcumin with encapsulation of PLGA and assessed them against prostate cancer cell lines, LNCaP, PC3, and DU145. The results exposed that the IC_50_ value of cells preserved with polymer-encapsulated nanocurcumin is less than the control group (Mukherjee and Vishwanatha, 2009). Numerous polymeric nanoformulations of curcumin were produced by crosslinking of N-vinyl-2-pyrrolidone, N-isopropyl acrylamide, and poly (ethylene glycol) monoacrylate and evaluate its antiproliferative activity against pancreatic cancer cells. The results revealed that polymer-loaded nanoparticles possess significant antiproliferative activity as compared to native curcumin (Bisht et al., 2007).

The anticancer activity was evaluated against breast cancer cell lines by stimulation of apoptosis using transferrin-mediated solid lipid nanoparticles (SLNs) of curcumin. The flow cytometric outcomes determined that the transferrin-mediated SLNs nanocurcumin has possibly elevated the anticancer activity of curcumin in breast cancer cell lines in *in vitro* studies compared to the standard group (Mulik et al., 2010). At the molecular level, the anticancer effects of curcumin underlie the mechanism of action by inhibiting the expression of the specificity protein (Sp) transcription factors Sp1, Sp3, and Sp4 (Meeta et al., 2017).

Sandhiutami et al. (2021) evaluated that the combination of curcumin and cisplatin nanoformulation with their similar effects may elevate the efficacy of therapeutic response and reduce the mortality of cancer patients. Wadhwa et al. (2014) optimized curcumin showed the IC_50_ value of 20.32 μM on the HT-29 colorectal cancer cell line which is a similar IC_50_ value of the prepared nanoformulation on HCT-116 cell lines.

A preliminary phytochemical examination of *Azadirachta indica* seed extract and isolated nimbin revealed the presence of a variety of phyto-constituents, including alkaloids, flavonoids, saponins, glycosides, polyphenols and tannins, steroids, and coumarins. It was discovered that the separated nimbin may include terpenoids, which may have enhanced the anticancer action.

In the present study, the IC_50_ value of the prepared nanosuspension was determined to be 30%. The nanosuspension dramatically and dose-dependently reduced the viability of cancer cells, as demonstrated by the *in vitro* MTT experiment. The curcumin–nimbin loaded nanosuspension produced promising anticancer action against HCT 116, it is further stressed. Further studies are required for the evaluation of the anticancer effect of curcumin–nimbin loaded nanosupension through clinical and preclinical studies for the development of potential formulation in the management of colorectal cancer.

## Conclusion

It can be concluded that curcumin–nimbin loaded nanosupension exhibited a potential anticancer activity against HCT 116 cell lines in both *in silico* and *in vitro* studies. Further studies are required to evaluate the anticancer effect of curcumin–nimbin loaded nanosuspension through clinical and preclinical studies for the growth of potential formulation in the management of colorectal cancer.
